# The relationship between peer victimisation, self-esteem, and internalizing symptoms in adolescents: A systematic review and meta-analysis

**DOI:** 10.1371/journal.pone.0282224

**Published:** 2023-03-29

**Authors:** Victoria M. R. Mullan, Dennis Golm, Jacob Juhl, Sana Sajid, Valerie Brandt

**Affiliations:** 1 School of Psychology, Centre for Innovation in Mental Health, University of Southampton, Southampton, United Kingdom; 2 Clinic of Psychiatry, Social Psychiatry and Psychotherapy, Hannover Medical School, Hanover, Germany; University of Study of Bari Aldo Moro, ITALY

## Abstract

**Background:**

Peer victimisation is common and predicts increased internalizing symptoms. Low self-esteem, which is associated with both greater peer victimisation and higher internalizing problems, may help explain why victimised adolescents experience greater internalizing symptoms. Objectives of the present research were to establish the relationships between peer victimisation, internalizing problems, and self-esteem, and to test whether self-esteem mediates the effect of victimisation on internalizing symptoms.

**Methods:**

We conducted a systematic literature search in Psychinfo, ERIC, Web of science, and Pubmed, following PRISMA guidelines. Inclusion criteria were: age 10–18 years; empirical studies that measured a) internalizing symptoms, b) self-esteem, and c) peer victimisation or bullying; design was either longitudinal or cross-sectional with a comparison group. Quality assessment were conducted using the Newcastle–Ottawa Quality Assessment Scale. We conducted random effects models and a meta-mediation analysis, with self-esteem acting as a mediator between peer victimization and internalizing symptoms.

**Results:**

Sixteen papers with a total of N = 35,032 (53% female) participants met the criteria for inclusion in the meta-analysis. The meta-analysis demonstrated an association between peer victimisation and both high internalizing problems (*r* = .31, CI 95 = .26 to.36) and low self-esteem (*r* = -.25, CI 95 = -.29; to -.22), and between low self-esteem and high internalizing problems ((*r* = -.38, CI 95 = -.42 to -.33), as well as an indirect effect of peer victimization on internalizing symptoms via self-esteem (ß = .10, CI lower = .07, CI upper = .13).

**Conclusions:**

Peer victimization, high internalizing symptoms and low self-esteem are all mutually related. Peer victimization partially mediates internalizing symptoms via self-esteem. Anti-bullying programmes may consider incorporating self-esteem building exercises in bully-victims. Limitations include high heterogeneity of results.

## Introduction

Globally, the prevalence of anxiety and depressive symptoms among adolescents is high. More than a third show elevated symptoms of depression [1] and 12% show elevated anxiety symptoms [2], which puts them at risk to develop a clinical presentation. It is therefore vital to identify modifiable risk factors and mechanisms for the development of these symptoms to identify targets for prevention.

Peer victimisation, defined as experiences of repeated maltreatment from one or more peers over time [3], constitutes one of these factors and is experienced by approximately 17% of children and adolecents [4], and this can have detrimental effects on mental health as well as affect a young persons’ self-esteem. Meta-analytic evidence points towards a causal relationship between experiences of peer victimisation and the development of internalising symptoms [5]. This relationship seems to be bi-directional however, with internalising symptoms also putting children at risk of victimisation [6,7].

The link between experiences of victimisation and the development of internalising symptoms could be explained by its impact on self-esteem. The development of self-esteem in children is influenced by the level of available social support through family and peers. Changes in the level of support are directly related to intra-individual changes of a child’s self-esteem [8]. This bi-directional relationship has been confirmed in a recent meta-analysis [9]. Bullying victimisation could indicate the absence of a good peer support network at school. Indeed, it has been shown that children who are continuously bullied have fewer friends at school [10].

While meta-analytic evidence confirms the negative association between bullying victimisation and self-esteem [11], a large longitudinal study in Chinese school children showed that lower self-esteem *mediated* the relationship between bullying victimation and internalising symptoms [12]. This would be consistent with the vulnerability model which assumes that low self-esteem contributes to depressive symptomatology [13].

Overall, current evidence suggests an interplay of victimisation experiences, lower self esteem and internalising symptoms. While meta-analyses have examined links between individual variables, no meta-analysis has been conducted which integrates all three variables of interest; bullying victimisation, self-esteem and internalising symptoms.

Our first aim was therefore to conduct a systematic review and meta-analysis to establish the relationship between peer victimisation, self-esteem and internalizing problems. Specifically, we aimed to investigate whether self-esteem is related to internalizing symptoms of adolescents aged between 10 and 18 who have experienced victimisation, and to gain an understanding of the relationship between victimisation and self-esteem and victimisation and internalizing symptoms independently. Our second aim was to assess whether self-esteem mediates the relationship between peer victimization and internalizing symptoms across studies.

Our first aim was to conduct a systematic review and meta-analysis to establish the relationship between peer victimisation, self-esteem and internalizing problems. Specifically, we aimed to investigate whether self-esteem is related to internalizing symptoms of adolescents aged between 10 and 18 who have experienced victimisation, and to gain an understanding of the relationship between victimisation and self-esteem and victimisation and internalizing symptoms independently. Our second aim was to assess whether self-esteem mediates the relationship between peer victimization and internalizing symptoms across studies.

## Methods

### Eligibility criteria

**Fig 1:**
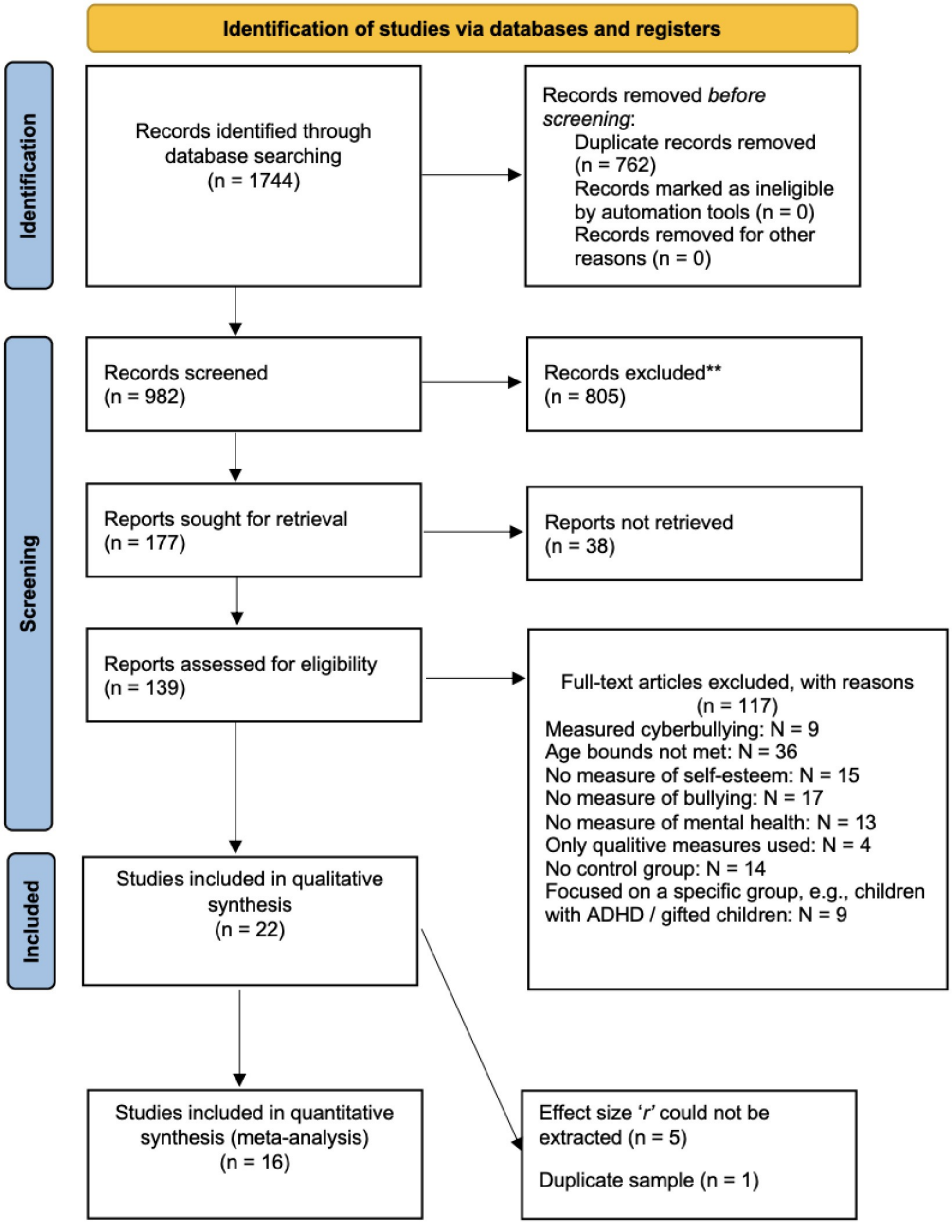
PRISMA diagram of the search process.

We conducted a systematic review following the preferred reporting items in systematic review and meta-analysis (PRISMA) guidelines (Fig 1) [14]. We screened all studies using pre-defined inclusion and exclusion criteria. Inclusion criteria were: age 10–18 years; empirical studies that measured a) internalizing symptoms (depression, anxiety), b) self-esteem, and c) peer victimisation or bullying; design was a) longitudinal or b) cross-sectional with a comparison group. We included studies conducted in languages other than English. Exclusion criteria were age > 18, no control group or longitudinal design, no standardised measures of internalizing symptoms and self-esteem, did not assess school bullying (e.g. cyberbullying).

### Search strategy

We conducted the systematic literature search across four electronic databases: Psychinfo, ERIC, Web of science, and Pubmed on August 15, 2022. We search the terms: ‘teen-age’, ‘young person’, ‘adolesc*’, ‘young adult’, ‘child*’, ‘self-esteem’, ‘self-concept’, ‘self-esteem’, ‘self-evaluation’, ‘self-perception’, ‘mental health’, ‘mentalillness’, ‘mental disorder’, ‘psychiatric illness’, ‘mental wellbeing’, ‘depression’, ‘anxiety’, ‘anxiety disorder’, ‘psychological distress’, ‘bully*’, and ‘victimi*’. We searched the references lists of included articles for additional relevant studies. V.M. and S.S. independently completed searches and screened the papers for inclusion/exclusion criteria.

### Study selection

After removing duplicates, 982 articles remained, which were screened independently by V. M. and S. S. Of these, 139 full-texts were screened. Finally, N = 22 papers were included in the qualitative synthesis. Two of these studies included the same sample [15,16]. Only the baseline measures have been published in a peer-reviewed journal and thus we only included baseline measures in the meta-analysis [16]. Five studies reported values that could not be transformed into *r* [17–21], and were therefore excluded from the meta-analysis. Therefore, 16 studies were included in the meta-analysis (Fig 1).

### Quality assessment

We conducted a quality assessment of the remaining 21 papers using an adapted version of the Newcastle–Ottawa Quality Assessment Scale for Cohort studies [22]. VM and SS conducted quality ratings independently, conlficts were later resolved by discussion with VB. This assessment was originally developed to measure the quality of non-randomised studies. We assessed quality with regard to a) the selection of the participants, b) the comparability of the groups, and c) the measurements used, i.e., whether the scales have been validated (e.g., standardised self-report and teacher reports, such as the Beck Depression Inventory [23], the Hospital Anxiety and Depression Scale [24], and the Social Anxiety Scale for Adolescents [25]). A rating of at least 4 out of 7 stars indicated acceptable quality. All papers had at least 4 stars (median = 5), therefore, no papers were excluded due to poor quality (see S1 Table for ratings of each paper).

### Statistical analysis

From each study, we extracted: characteristics of the sample (i.e., country where the study was conducted, sample size, age and sex ratio [where available]) and the measure victimisation, self-esteem, and internalizing symptoms (Table 1). Data was extracted by VM and VB independently and compared. Conflicts were resolved by discussion. We report *r* as a measure of effect size (see Table 1). If *r* was not originally reported, it was calculated it from means and standard deviations where possible. Where papers reported correlations at more than one time point, we averaged across these correlation for the purpose of the meta-analysis. Not all studies assessed the association between self-esteem and internalising symptoms or only reported cross-sectional correlations for those two variables, leading to fewer studies veing included regarding the relationship between self-esteem and internalizing symptoms, and therefore the meta-mediation was restricted to 7 studies (N = 17380).

**Table 1:** Details of 22 included studies.

	First Author	Sample	Year	N	Age	F%	Victimisation	Outcome		Results	Victimisation/Internalizing symptoms		Self-esteem/Internalizing
		Country (design)					Measure	Self-esteem measure	Internalizing symptoms measure	Victimisation /Self-esteem	Anxiety	Depression	
1	Bogart	USA (long)	2014	4297	10–16 M = 11.1 (0.6)	51	Peer experience questionnaire (Felix, Sharkey, Green, Furlong, & Tanigawa, 2011) [26]	Self-perception questionnaire (Mussen & Hetherington, 1983) [27]	Depression subscale of the Diagnostic Interview Schedule for Children Predictive Scales (Lucas, Zhang, & Fisher, 2001) [28]	Covariate-adjusted standardized mean = -.60	-	Covariate-adjusted standardized mean = .79	-
2	Borg	Canada (long)	2021	1072	10–16 M = 12.5 (1.71)	50	Peer Victimization scale (Daly & Willoughby, 2020) [29]	Self-esteem Rosenberg (1965)	Depression scale for children (Weissman, Orvaschel, & Padian, 1980) [30]	r = -.20	r = .17	r = .16	
3	Estévez	Spain (cross)	2009	1319	11–16 M = 13.7 (1.6)	53	Peer Victimisation Scale (Maynard & Joseph, 2000) [29]	Self-esteem Rosenberg (1965)	Centre of Epidemiological Studies Depression Scale (Radloff, 1977)	r = -.25	-	r = .30	-
4	Evans	USA (long)	2018	8000	11–18 M = 12.5	51	School Success Profile + (Bowen & Richman, 2008) [31]	Self-esteem Rosenberg (1965)	Youth Self-report (YSr) (Achenbach, 1991) [32]	r = -.18	r = .23		r = -.33
5	Graham	USA (cross)	2003	775	11–12 M = 11.5	56	Peer Victimsation Scale (Neary & Joseph, 1994)	Global self-esteem subscale (Harter Self-Perception Profile for Children)	Social Anxiety Scale for Adolescents (La Greca & Lopez, 1998) [33] Short form of the Children’s Depression Inventory (Kovacs, 1985) [34]	Standardised mean difference = -.63	Standardised mean difference = .64	Standardised mean difference = .84	-
6	Grills	USA (cross)	2002	279	11–12 M = 11.8 (.53)	53	Peer Victimisation Scale (Maynard & Joseph, 2000)	Self-perception Profile for Children (Harter, 1985) [35]	Multidimensional Anxiety Scale (March, 1997) [36]	(M) r = -.55 (F) r = -.33	(M) r = .26 (F) r = .19	-	(M) r = -.19 (F) r = -.29
7	Grills	USA (long)	2003	77	11–15 M = 13.6 (.60)	52	Peer Victimisation Scale (Maynard & Joseph, 2000)	Self-perception Profile for Children (Harter, 1985) [35]	Multidimensional Anxiety Scale (March, 1997) Reynold’s Adolescent Depression Scale	(M) r = .34 (F) r = .47	(M) r = -.09 (F) r = -.25	(M) r = -.36 (F) r = -.21	(M anx/depr) r = -.26 / -.56 (F anx/depr) r = -.26 / -.42
8	Hesapçıoğlu	Turkey (cross)	2018	1173	15–18	-	Peer Bullying Questionnaire (Piskin, 2002)[37]	Coopersmith Self-Esteem Scale (Coopersmith, 1981) [38]	Beck Depression Inventory (Beck, Ward, Mendelson, Mock, & Erbaugh, 1961) [39]	r = -.22	-	r = .22	-
9	Juvonen	USA (cross & long)	2000	243	12–15	55	Peer Victimsation Scale (Neary & Joseph, 1994)	Self-perception Profile for Children (Harter, 1985) [35]	Children’s Depression Inventory (Kovacs, 1985) [34]	r = -.24	-	r = .17	r = -.69
10	Låftman	Sweden (cross)	2017	4319	14–15 M = 14.8 (.50)	52	Researcher developed questions	Researcher developed questions. Model adjusted for: sex, age, family type, immigrant background, parent social class	Researcher developed questions. Model adjusted for: sex, age, family type, immigrant background, parent social class	OR = -.45	b = .73		-
11	Marini	Canada (cross)	2006	7290	13–18 M = 15.6 (1.3)	52	Bullying behavioural checklist (Marini, 1998). Here included: direct bullying victims vs. uninvolved	Self-esteem Rosenberg (1965)	Social anxiety measure–adaptation of Ginsburg et al (1998) Centre for Epidemiological Studies Depression Scale (National Institute of Mental Health, USA, 1972)	r = -.24	r = .22	r = .23	r = -.27 (anx) r = -.55 (depr)
12	McVie	Scotland (long)	2014	4300	13–17 Longitudinal	51	Adapted Olweus Bully/Victim Questionnaire (Olweus, 1993) [40]	Self-esteem Rosenberg (1965)	Hospital Anxiety and Depression Scale (HADS)	ß = -.65	ß = 1.00	-	
13	O’ Moore	Ireland (cross)	2001	5797	12–18 Longitudinal	63	Olweus Self-Report Questionnaire (Olweus, 1993) [40]	Piers-Harris Self-Concept Scale (Piers 1984)	Piers-Harris Self-Concept Scale (Piers 1984)—Anxiety	F = .24	F = .24	-	-
14	Saint-Georges	Canada (long)	2020	612	12–16 M = 12.4	54	Olweus Bully/Victim questionnaire (Vaillancourt et al, 2010)	Behavior Assessment System for Children– 2^nd^ edition	Behavior Assessment System for Children– 2^nd^ edition	r = -.27 Averaged across grades 8–11	N/A	r = .28 Averaged	r = -.41 Averaged
15	Sapouna	UK (long)	2013	3136	13–14 assessed at age 13 and 14	52	Olweus Self-Report Questionnaire (Olweus, 1993) [40]	Self-esteem Rosenberg (1965)	West Scotland 11–16 study of Teenage Health and Depression	r = -.14	N/A	r = .28	r = -.27
16	Seals	USA (cross)	2003	1126	12–14	59	Peer Relations Questionnaire (Rigby & Slee, 1995) [41]	Self-esteem Rosenberg (1965)	Children’s Depressive Inventory (Kovacs, 1985) [34]	r = -.07	N/A	r = .44	-
17	Sharpe	UK (long)	2020	13917	11–14	49	Item 1: “How often other children hurt or pick on you on purpose” Item 2: “How often brothers or sisters hurt or pick on you on purpose”	Self-esteem Rosenberg (1965)	Age 11: Asked “How often in the past 4 weeks they felt “sad”, “afraid or scared” and “worried about what would happen” Age 14: Moods and Feelings Questionnaire (MFQ) (Angold, 1989) 66]	(M) ß = -.29 (F) ß = -.48	-	(M) ß = .78 (F) ß = .89	-
18	Soler	Spain (cross)	2013	736	14–18 M = 15.7 (1.2)	63	Juvenile Victimisation Questionnaire (Hamby, Finkelhor, Ormorod, & Turner, 2004)	Self-esteem Rosenberg (1965)	Youth Self Report (Achenbach & Rescorla, 2001)	(M) r = -.25 / -.19 (F) r = -.18 / -.11	(M) r = .43 (F) r = .31	(M) r = -.62 / -.21 (F) r = -.54 / -.34	
19	Tennant	USA (cross)	2019	700	10–13	49	BPBQ (Summers & Demaray, 2008)	RSES (Rosenberg, 1965)	SCARED (Birmaher et al, 1999) CES–D (Radloff, 1977)	(M) r = -.26 (F) r = -.45	(M) r = .33 (F) r = .40	(M) r = .46 (F) r = .59	(M) r = -.31 /-.17 (F) r = -.51 / -.45
20	Undheim	Norway (cross)	2010	2464	12–15 M = 13.7 (0.6)	51	Researcher developed questions	Harter’s Self-Perception Profile for adolescents (Harter, 1985) [35]	Moods and Feelings Questionnaire (MFQ) (Angold, 1989) [42]	r = -.31	r = .46	-	
21	Wang	USA (long)	2011	1171	10–17 M = 12.2 (1.3)	53	Social Experiences Questionnaire (Crick & Grotpeter, 1996) [43]	Self-Descriptive Questionnaire (Marsh, 1989) [44]	Children’s Depression Inventory (Kovacs, 1985) [34] Multidimensional Anxiety Scale for Children (March, 1997)	-	standardized loading = 0.26 (relational) / -.08 (overt)	standardized loading = 0.33 (relational) / -.01 (overt)	standardized loading = -.22 (depr) / -.13 (anx)
22	Ybrandt	Sweden (cross)	2010	204	12–16 M = 13.9	48	Social problems subscale (YSr) (Achenbach, 1991) [32]	Self-esteem = I think I am’ (ItIA) (Ouvinen-birgerstam, 1999) [45]	Youth Self-report (YSr) (Achenbach, 1991) [32]	r = -.35	r = .41	-	r = -.53

We computed weighted summary measures for the effects of victimisation on self-esteem and internalizing symptoms, and for the association between self-esteem and internalizing symptoms, using Comprehensive Meta-Analysis software [46]. Some studies reported effects sperately for males and females, and where this was the case, we also included male and female samples separately. Synthesised effects are reported as *r* with 95% Confidence intervals (CI). The Eggers test was used to explore possible publication bias.

Sensitivity analyses were conducted excluding studies reporting statistic other than *r* or mean and SD, and one study excluding non-victims from their final analysis. Heteroneheity due to mean age (N = 11 studies) was explored using meta-regression in the relationship between peer victimization and self-esteem and peer victimization and internalizing symptoms. Not enough studies could be included to conduct a meta-regression for the relationship between self-esteem and internalizing symptoms. Not enough studies reported results by gender, so that no meta-regression was possible using gender.

A meta-mediation model was conducted in R, using the metaSEM package [47], where self-esteem mediated the relationship between peer victimization and internalizing symptoms (N = 7 studies included). All meta-analyses were conducted using random-effect models to account for heterogeneity in the effect sizes between studies.

## Results

The analysis comprised N = 35,032 participants (53% female, sex data available from 15 studies) from N = 16 studies that were included in the meta-analysis. The age ranged from 10–18 (Mean = 13.39; data available from 13 studies). Sample size from these studies ranged from 60–8000. Six out of the 15 studies were longitudinal [48–52].

Seven studies assessed depression only, one study assessed anxiety only, five studies assessed depression and additional constructs, such as anxiety, social anxiety or loneliness. Four studies assessed internalizing symptoms (Table 1). Eight studies made group comparisons, nine studies assessed peer victimisation continuously, one of these studies assessed cumulative peer victimisation over time. One study encompassed a non-victimised group but excluded the group from their analysis; this study was removed for the sensitivity analysis. Eight studies reported correlations, six studies reported means and standard deviations, one study reported odds ration for the relationship between victimisation and self-esteem and standardised means for the relationship between internalizing symptoms and victimisation.

### Victimisation, self-esteem, and internalizing symptoms

**Fig 2:**
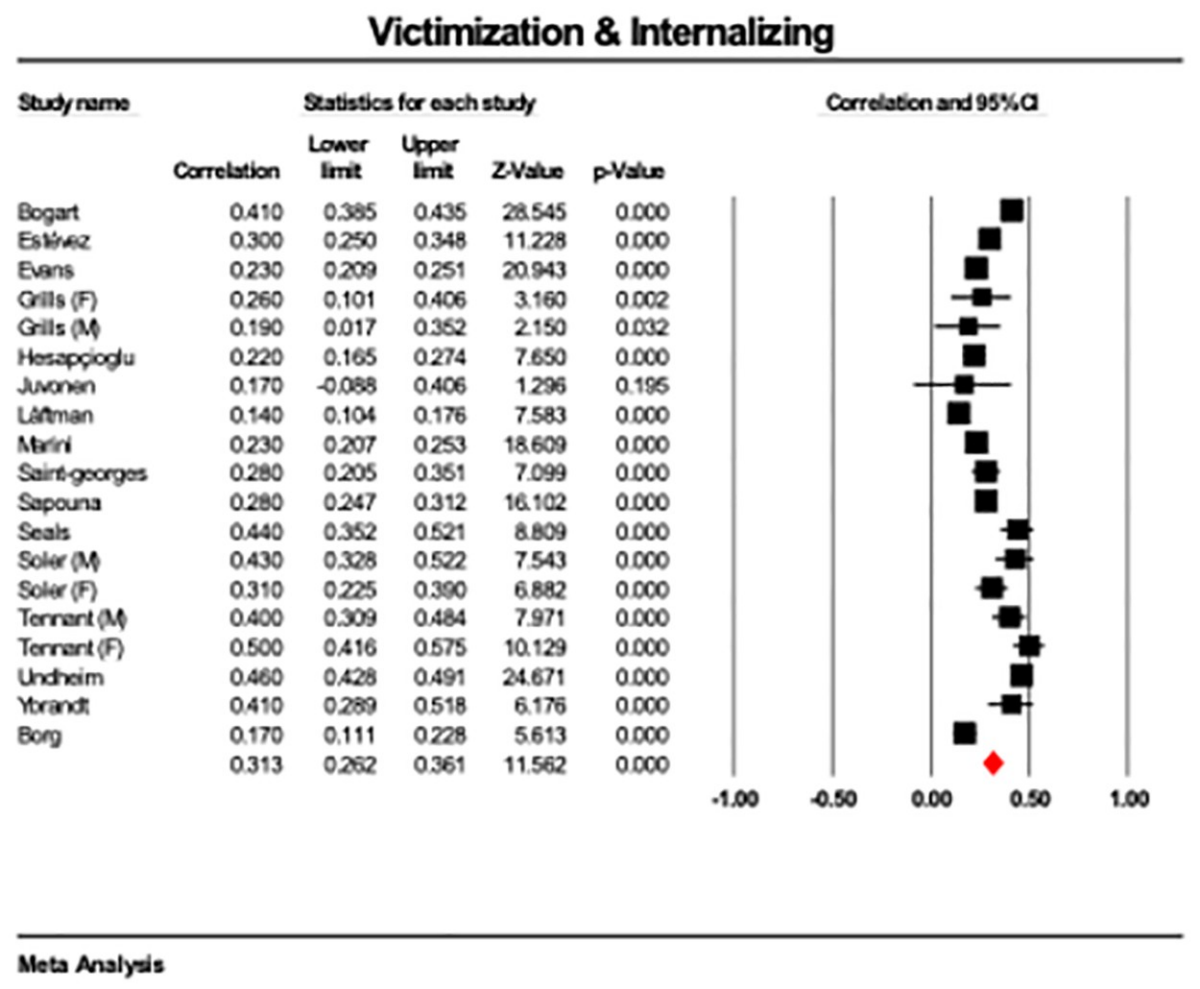
Forest plot showing correlations between victimisation and internalising symptoms across 15 studies with 18 reported effect sizes, with overall effect highlighted in red.

The meta-analysis showed that victimization was significantly associated with higher internalizing symptoms across studies (*r* = .31, CI 95 = .26 to.36, p < .001; Fig 2). The results were highly heterogeneous (Q = 381.63, p < .001, I^2^ = 95). The Egger’s test was not significant (*p* = .343), indicating that the funnel plot is symmetrical and publication bias is unlikely to account for the results (S1 Fig). A meta-regression entering mean age as a covariate for those studies that reported mean age showed a non-significant result (ß = .003, CI95 = -.04 to.05, p = .880).

**Fig 3:**
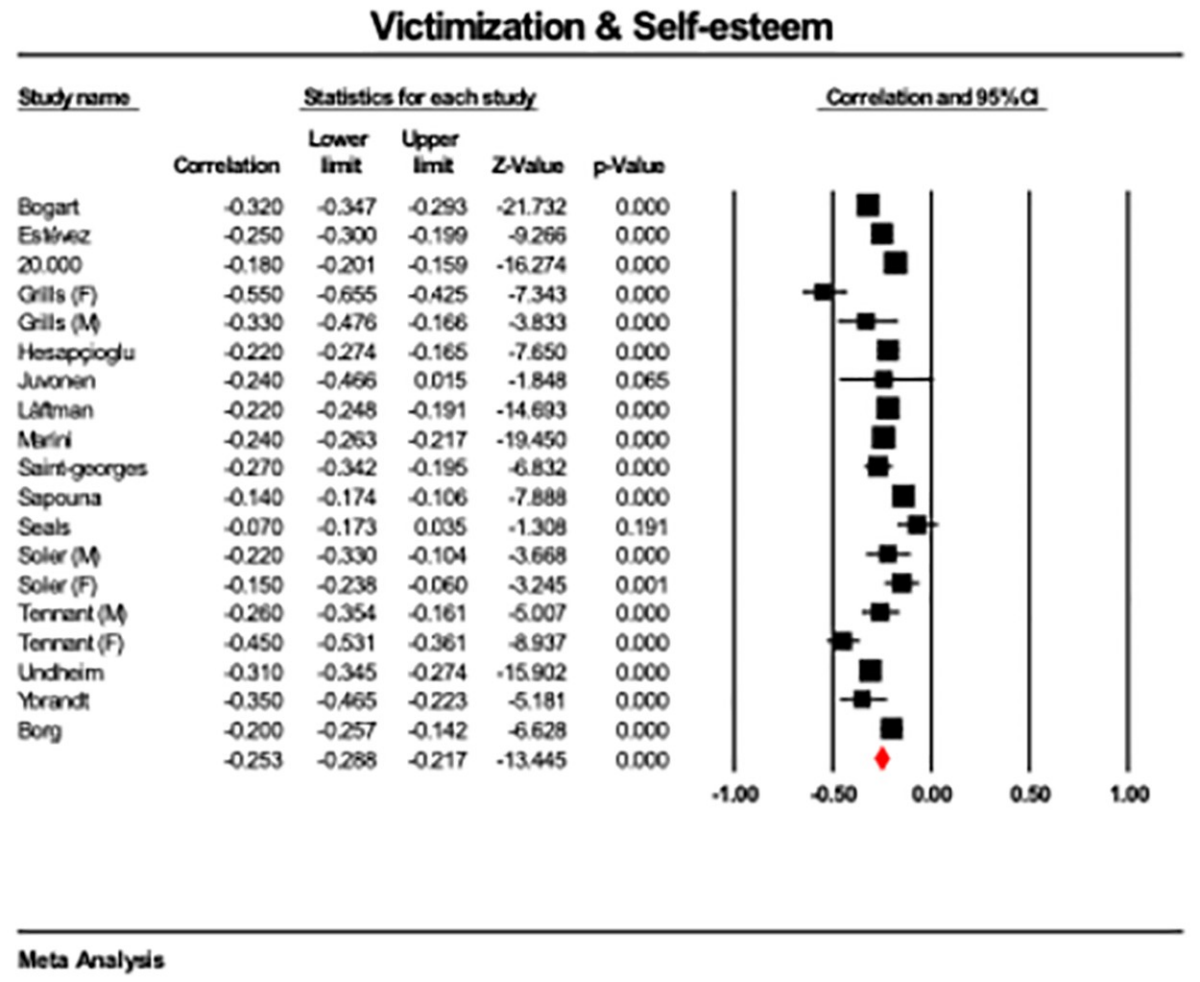
Forest plot showing correlations between victimisation and self-esteem scores across 15 studies with 18 reported effect sizes, with overall effect highlighted in red.

Victimization was moderately associated with lower self-esteem across studies (*r* = -.25, CI 95 = -.29; to -.22, *p* < 0.001; Fig 3). The results were highly heterogeneous (Q = 172.24, p < .001, I^2^ = 90), while the Egger’s test was not significant (*p* = .281; S2 Fig).

A meta-regression entering mean age as a covariate for those studies that reported mean age showed a non-significant result (ß = .03, CI95 = -.01 to.01, p = .106).

**Fig 4:**
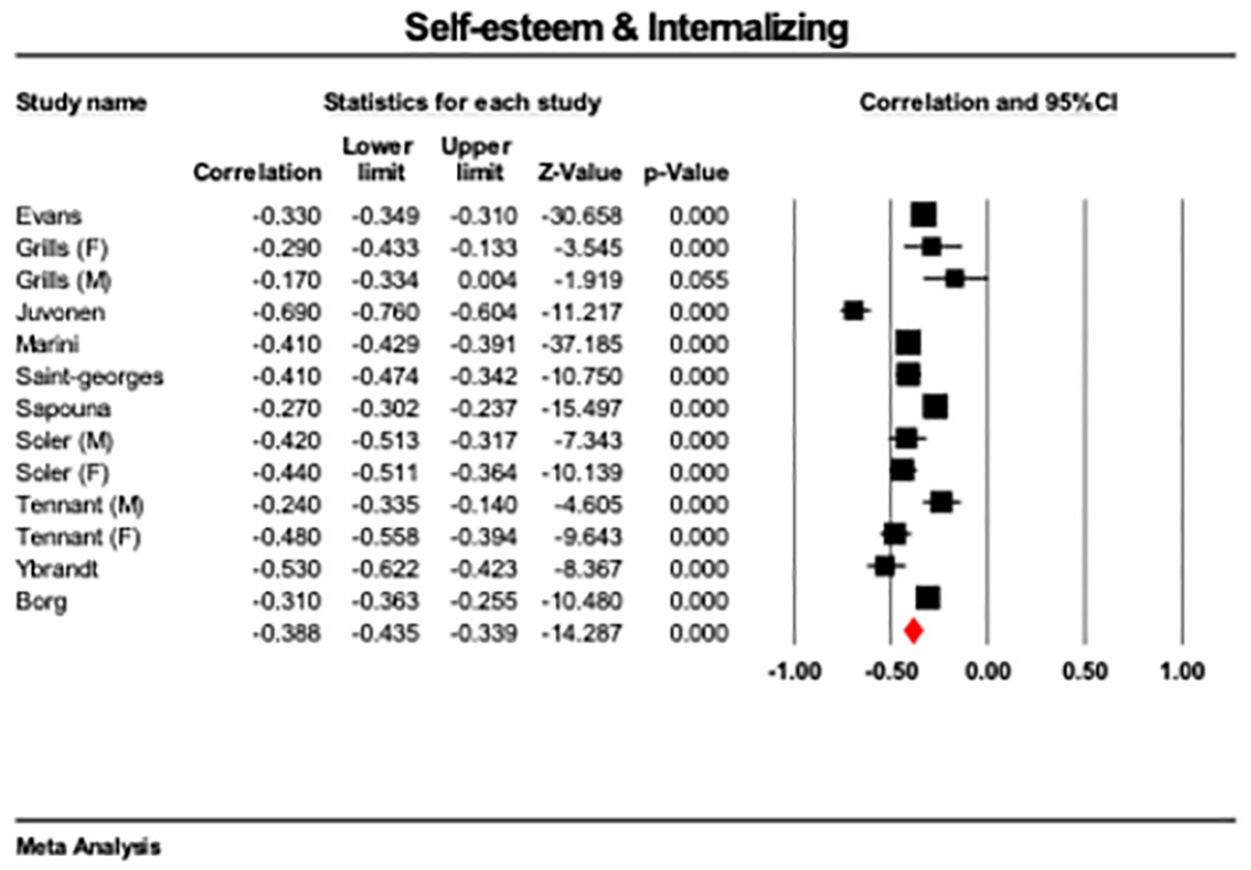
Forest plot showing correlations between internalizing symptoms and self-esteem scores across 7 studies with 10 reported effect sizes, with overall effect highlighted in red.

Lower self-esteem was associated with higher internalizing symptoms across studies (*r* = -.38, CI 95 = -.42 to -.33, *p* < 0.001, Fig 4) The results were highly heterogeneous (Q = 113.85, p < .001, I^2^ = 89) while the Egger’s test was not significant (*p* = .498; S3 Fig). A meta-regression entering mean age as a covariate for those studies that reported mean age showed small, significant result (ß = -.04, CI95 = -.07 to -.01, p = .007).

**Fig 5:**
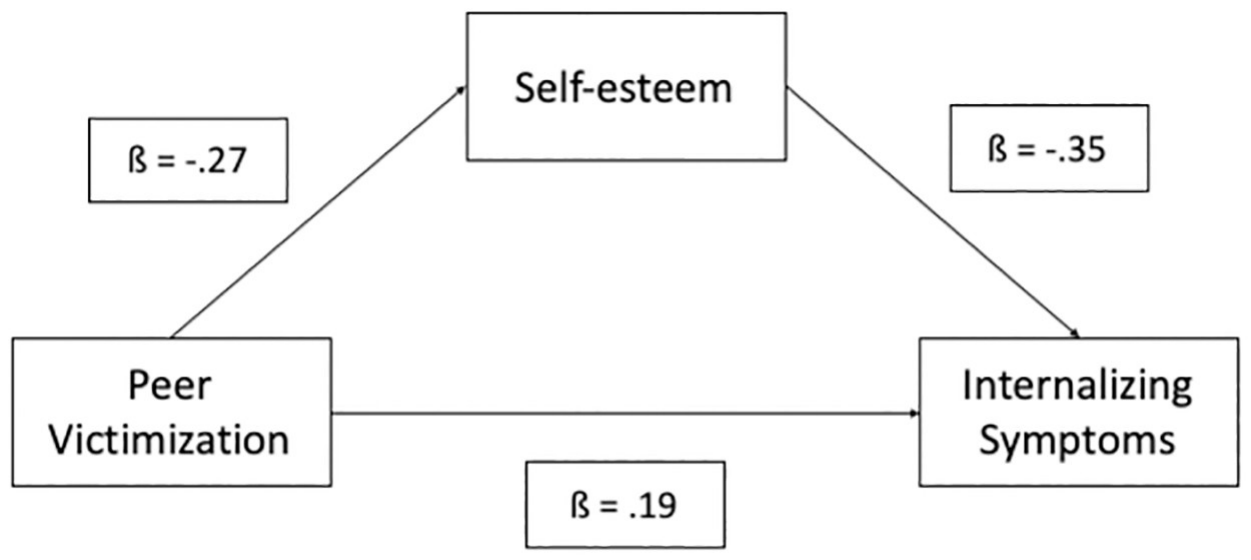
Peer victimization was associated with self-esteem and internalizing symptoms and self-esteem was associated with internalizing symptoms. In addition, peer victimization was associated with internalizing symptoms indirectly via self-esteem.

The meta-mediation model showed a significant indirect effect of peer victimization on internalizing symptoms via self-esteem ß = .10, CI lower = .07, CI upper = .13; for direct effects see Fig 5).

### Gender

Only three studies reported results by gender. Entering gender in a meta-regression showed non-significant results for the relationship between victimization and self-esteem (ß = -.13, CI95 = -.43 to.17, *p* = .382), peer victimization and internalizing symptoms (ß = -.01, CI95 = -.21 to.24, *p* = .898), and self-esteem and internalizing symptoms (ß = -.15, CI95 = -.34 to.04, *p* = .126).

### Sensitivity analysis

The sensitivity analysis showed that the results were robust (S4–S6 Figs).

## Discussion

The results of this meta-analysis show that individuals who have been victimised by their peers have higher internalizing problems and lower self-esteem, while lower self-esteem is also associated with higher internalizing problems. Additionally, a small but significant indicrect effect showed that self-esteem mediates the relationship between peer victimization and internalizing symptoms. The results clarify that one way in which bullying can impact internalizing symptoms is by affecting self-esteem, although the size of the effect indicates that other factors might play a role as well. The findings confirm original research pointing to self-esteem as a mediator of the effect of peer victimization on depression and anxiety [12,15–17,53,54].

The finding that peer victimization and internalizing symptoms are closely linked has been recognized in a wealth of literature [15,52,55,56] and is in line with findings from other meta-analyses showing that peer victimization has a small effect on later internalizing symptoms and that young people with internalizing symptoms are at high risk to experience peer victimization, irrespective of age and gender [6,56]. School programms addressing peer victimization have been shown to have a small to moderate positive effect [57]. The most promising programmes involved the establishment of an anti-bullying school policy and elements of peer counselling and emotional control [57].

Our results are congruent with previous meta-analyses that showed a small effect of peer victimization on internalizing symptoms [6], however, the results showed that the direct effect of peer victimization on self-esteem was larger than on internalizing symptoms, with a medium effect size. This is important to considers. Self-esteem, in turn, has an effect on social relationships [9], mental, and physical health [58]. A medium relationship between self-esteem and internalizing symptoms was also reflected in the results of this meta-analysis.

The direct effect between self-esteem and dinternalizing symptoms is consistent with the vulnerability model of depression, which highlights low-self-esteem as a risk factor for the development of depression [13]. However, the vulnerability model might be too simplistic as it does not consider the influence of shared risk factors or mediators. Our results are also consistent with the Identity Disruption Model [59], which assumes that adverse experiences are associated with negative mental health outcomes through disruptions of identity development (i.e., an unclear/ unstable sense of self). While this meta-analysis did not focus on the concept of self-concept clarity, this variable is highly correlated with self-esteem [60] and might be affected by peer victimization.

Our results highlight bullying victimization and low self-esteem as possible targets for intervention or prevention approaches. Most anti-bullying programmes focus on reducing aggressive acts by installing consequences, removing the reinforcing factors on the environment (e.g. peers as spectators), and to intervene in bullying acts immediately. Some programmes involve talks with bullies, victims, and their parents, and assertiveness traiuninfgwith the bully-victim [61–63]. Overall, the focus seems to be on perpetration. Our results suggest that it might be useful to include interventions targeting the bully-victims as well as the perpetrators. While it might be difficult to treat internalizing symptoms directly, bully-victims may benefit from programme elements that help them recognize the effects bullying can have on self-esteem and help bully-victims build or re-build self-esteem, not only assertiveness. Our results indicate that the relationship between low self-esteem and internalizing symptoms became weaker with higher age, suggesting that interventions would be particularly important for younger children.

Evidence for the effectiveness for this type of interventions stems from research in adults: A cognitive-behavioral group intervention that specifically targeted self-esteem, the Overcoming Low Self-Esteem Intervention, demonstrated an increase in self-esteem, and a decrease in internalizing symptoms at the three months follow-up in adults [64]. Future research might assess whether building self-esteem in bully-victims might reduce both internalizing symptoms and the risk of future peer victimization. Implementation of similar programmes for the school context might be promising. The UK government recently established the role of Education Mental Health Practitioners, who deliver low-intensity cognitive-behavioral therapy within a school context [65]. While the role focuses on the treatment of mental health problems, the evidence from this meta-analysis would also support the establishment of self-esteem building groups in schools that could be led by people in this specialized role.

## Strengths and limitations

Our research had a number of strengths. This was the first comprehensive meta-analysis to simultaneously explore the associations between peer victimisation, self-esteem and internalizing problems. Further, only longitudinal studies or studies with a control group were included in the analyses and the majority of studies investigated large groups of participants.

Cyberbullying was not included into the analysis as a focus was put on bullying that occurred in schools. However, cyberbullying is related to self-esteem and internalizing symptoms in a similar way. A cross-sectional study on Vietnamese students demonstrated that self-esteem mediated the relationship between experiencing cyberbullying and depression symptoms [66], and a study on Italian adolescents found that lower self-esteem was related to a greater risk of being cyberbullied [67]. Moreover, a longitudinal study on Chinese adolescents found that cyberbullying positively predicted internalizing problems [68].

A further weakness of this study is that is was not possible to pinpoint the source of heterogeneity in the results. Only three studies reported results by gender, therefore, even though the results were not significant, it would be difficult to interpret the absence of a significant effect for so few studies. Gender can affect the relationship between peer victimization and internalizing symptoms [69], however, we could not show that the mediation via self-esteem was affected by gender. Given the high heterogeneity, results should be interpreted with caution. It should however be pointed out that almost all results across all outcomes were significant and change occurred in the same direction. While the high heterogeneity makes it harder to determine the true effect size, there is no doubt that there is a significant relationship between the variables. Lastly, this review was not registered and we did not write a protocol.

## Conclusions

Our meta-analysis showed that peer victimisation poses a risk factor for developing low self-esteem and internalizing problems, and that peer victimization increases internalizing problems partially via lower self-esteem, although with a small effect size.

## Supporting information

S1 Checklist PRISMA 2020 checklist. (DOCX)

S1 Fig Funnel plot for the relationship between victimization and internalizing symptoms. The eggers test was non-significant, indicating that a publication bias is unlikely to account for the results.
(TIF)

S2 Fig Funnel plot for the relationship between victimization and self-esteem. The eggers test was non-significant, indicating that a publication bias is unlikely to account for the results.
(TIF)

S3 Fig Funnel plot for the relationship between self-esteem and internalising symptoms. The eggers test was non-significant, indicating that a publication bias is unlikely to account for the results.
(TIF)

S4 Fig The sensitivity analysis shows that the association between victimisation and internalising symptoms remains significant. (TIF)

S5 Fig The sensitivity analysis shows that the association between victimisation and Self-Esteem remains significant. (TIF)

S6 Fig The sensitivity analysis shows that the association between internalising symptoms and self-esteem remains significant. (TIF)

S1 Table Shows the subdomains of quality ratings and the overall number of stars each included paper received (7 stars maximum). (DOCX)
