# Supplementary figures and images for: The relationship between peer victimisation, self-esteem, and internalizing symptoms in adolescents: A systematic review and meta-analysis
Source: PLoS One. 2023 Mar 29;18(3):e0282224. doi: 10.1371/journal.pone.0282224 (PMC10058150; doi:10.1371/journal.pone.0282224)

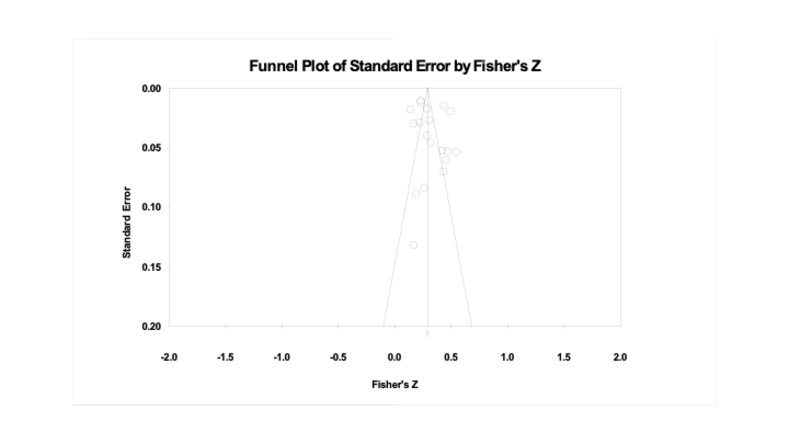

Supplement: S1 Fig — The eggers test was non-significant, indicating that a publication bias is unlikely to account for the results. (TIF) [file pone.0282224.s002.tif]

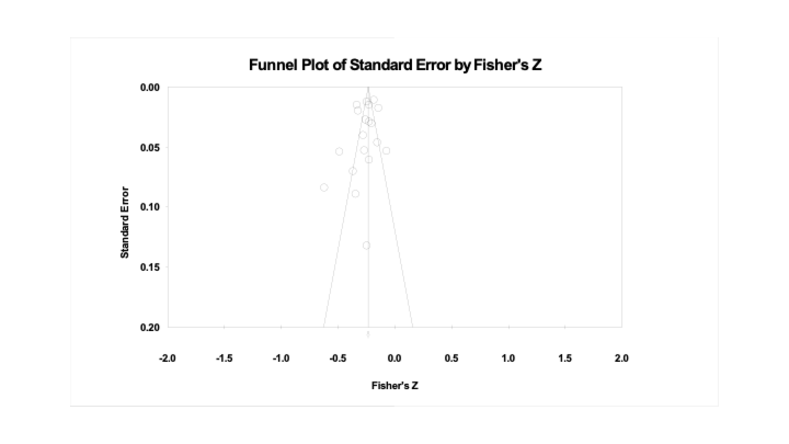

Supplement: S2 Fig — The eggers test was non-significant, indicating that a publication bias is unlikely to account for the results. (TIF) [file pone.0282224.s003.tif]

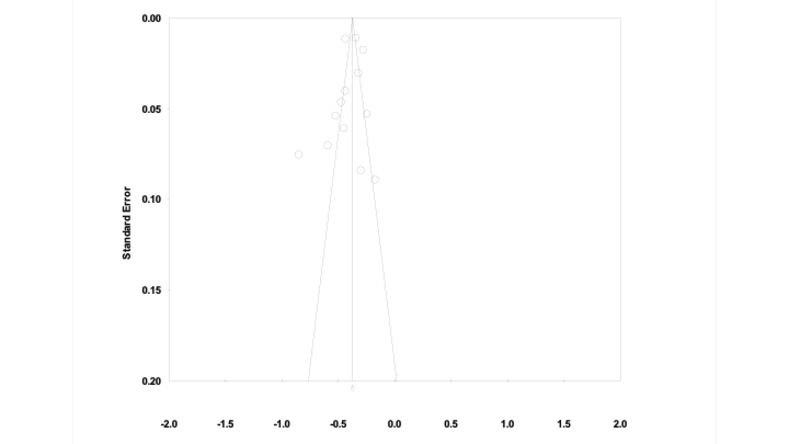

Supplement: S3 Fig — The eggers test was non-significant, indicating that a publication bias is unlikely to account for the results. (TIF) [file pone.0282224.s004.tif]

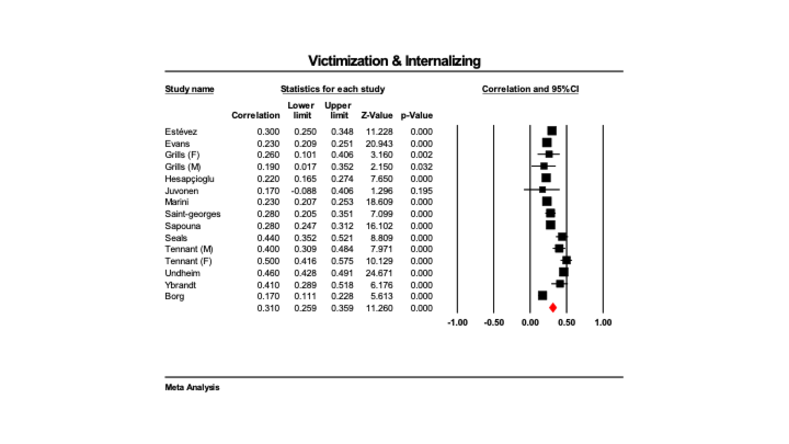

Supplement: S4 Fig — (TIF) [file pone.0282224.s005.tif]

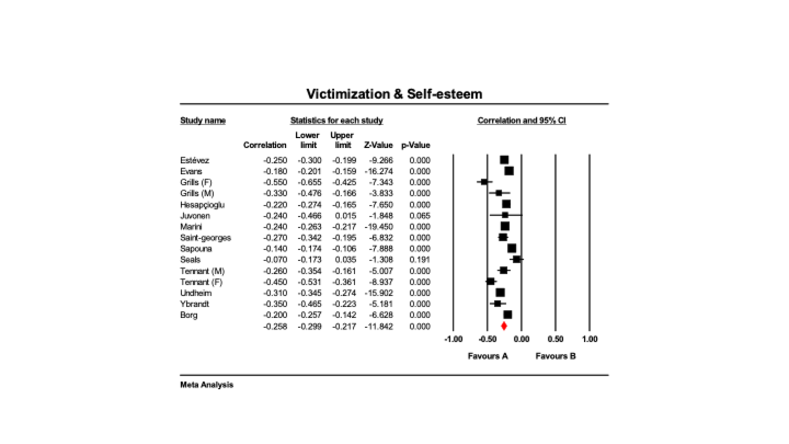

Supplement: S5 Fig — (TIF) [file pone.0282224.s006.tif]

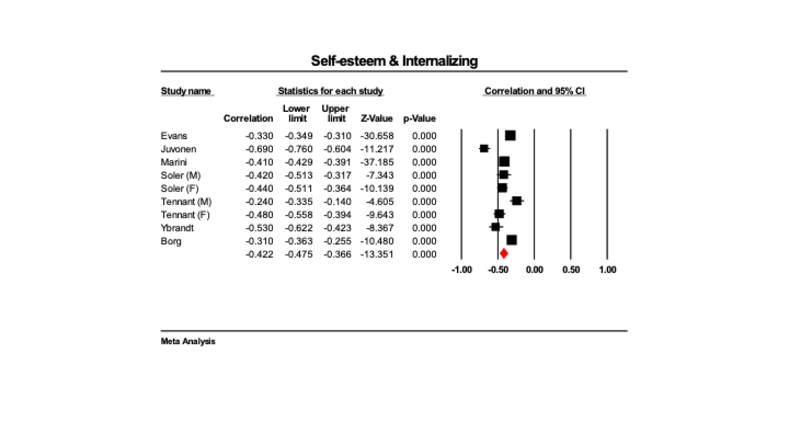

Supplement: S6 Fig — (TIF) [file pone.0282224.s007.tif]
