# Supplementary material for: The relationship between peer victimisation, self-esteem, and internalizing symptoms in adolescents: A systematic review and meta-analysis
Source: PLoS One. 2023 Mar 29;18(3):e0282224. doi: 10.1371/journal.pone.0282224 (PMC10058150; doi:10.1371/journal.pone.0282224)
Supplement: S1 Table — (DOCX) [file pone.0282224.s008.docx]

Supplementary Table S1

| First Author | Representativeness of cohort | Selection of non-exposed cohort | Ascertainment of Exposure | Comparability | Assessment of outcome | Follow up | Adequacy of  follow up | Total |
| --- | --- | --- | --- | --- | --- | --- | --- | --- |
| Bogart | * | ***** | ***** | ***** | ***** | X | X | 5 |
| Estévez | ***** | ***** | ***** | ***** | ***** | X | X | 5 |
| Evans | ***** | ***** | ***** | ***** | ***** | ***** | X | 6 |
| Graham | X | ***** | ***** | ***** | ***** | X | X | 4 |
| Grills | X | ***** | ***** | ***** | ***** | X | X | 4 |
| Grills (2003) | X | ***** | ***** | ***** | ***** | ***** | X | 4 |
| Hesapçıoğlu | ***** | ***** | ***** | ***** | ***** | X | X | 5 |
| Juvonen | X | ***** | ***** | ***** | ***** | ***** | X | 6 |
| Låftman | ***** | ***** | X | ***** | X | X | X | 3 |
| Marini | ***** | ***** | ***** | ***** | ***** | X | X | 5 |
| McVie | ***** | ***** | ***** | ***** | ***** | ***** | ***** | 7 |
| O’Moore | ***** | ***** | ***** | ***** | ***** | X | X | 5 |
| Saint-Georges | ***** | ***** | ***** | ***** | ***** | ***** | ***** | 7 |
| Sapouna | ***** | ***** | ***** | ***** | ***** | ***** | ***** | 7 |
| Seals | ***** | ***** | ***** | ***** | ***** | X | X | 5 |
| Sharpe | ***** | ***** | X | ***** | ***** | ***** | ***** | 6 |
| Soler | X | ***** | ***** | ***** | ***** | X | X | 5 |
| Tennant | X | ***** | ***** | ***** | ***** | X | X | 4 |
| Undheim | ***** | ***** | X | ***** | ***** | X | X | 4 |
| Wang | ***** | ***** | ***** | ***** | ***** | X | X | 5 |
| Ybrandt | X | ***** | ***** | ***** | ***** | X | X | 5 |

Supplementary Table S1 shows the subdomains of quality ratings and the overall number of stars each included paper received (7 stars maximum).
